# Beyond multi view deconvolution for inherently aligned fluorescence tomography

**DOI:** 10.1038/s41598-021-95266-2

**Published:** 2021-08-03

**Authors:** Daniele Ancora, Gianluca Valentini, Antonio Pifferi, Andrea Bassi

**Affiliations:** 1 Dipartimento di Fisica, Politecnico di Milano, Piazza Leonardo da Vinci 32, 20133 Milan, Italy; 2 Istituto di Fotonica e Nanotecnologie, Consiglio Nazionale delle Ricerche, Piazza Leonardo da Vinci 32, 20133 Milan, Italy

**Keywords:** Applied optics, Optical physics, Information theory and computation, Imaging and sensing, Optical techniques, Light-sheet microscopy

## Abstract

In multi-view fluorescence microscopy, each angular acquisition needs to be aligned with care to obtain an optimal volumetric reconstruction. Here, instead, we propose a neat protocol based on auto-correlation inversion, that leads directly to the formation of inherently aligned tomographies. Our method generates sharp reconstructions, with the same accuracy reachable after sub-pixel alignment but with improved point-spread-function. The procedure can be performed simultaneously with deconvolution further increasing the reconstruction resolution.

## Introduction

The field of tomographic imaging experienced a silent revolution during the last decade. A strong demand driven by deep learning and data mining has prompted hardware manufacturers to improve computing performances while keeping the price affordable. Nowadays, high throughput computation is possible with graphic processing units (GPU). GPUs allow parallel data-processing with performances beyond belief just a few years ago, radically changing the field of signal processing. In particular, standard image processing tasks such as Fourier-transformation, convolution, and matrix operations experience a constant-rate performance increase^1,2^. GPUs are the ideal solution for the massive image processing tasks required by tomographic reconstructions^3^. At visible wavelengths, optical projection tomography (OPT) is an example of an imaging technique applied for tomographic studies at microscopic level^4^. By rotating the specimen and collecting its optical projections at multiple angles, it is possible to form the reconstruction of the specimen via tomographic inversion. Another optical technique, light-sheet fluorescence microscopy (LSFM), offers a straightforward way to optically section the sample for the inspection of its internal structure^5^. Even if LSFM is a direct tomographic technique (i.e., it does not strictly require computation to generate a section of the sample) it is often desirable to observe the object from different angles to increase the reconstruction quality^6^. LSFM suffers from non-isotropic resolution (the axial resolution is lower than the lateral) and, in many cases, the sample is not visible as a whole due to tissue scattering or absorption. Multi-view approaches address these problems, either relying on the sample rotation^7^ or exploiting multiple objectives to observe the specimen from different angles^8^. Before their fusion, each acquisition is registered (aligned) against a chosen reference^9^, to place the information captured at different angles appropriately. Usually, the registration is accomplished by locating the best overlap between the volumes, eventually including beads around the specimen to enforce the alignment fidelity^10^. Here we discuss a new reconstruction strategy for the formation of an inherently aligned tomographic view of a biological specimen. We exploit the property of the auto-correlation (we indicate it by the operator ${\mathcal {A}}$) to avoid any alignment procedure. At the same time, we demonstrate that the reconstruction based on multi-view auto-correlation brings an improved resolution due to the rejections of second order-correlations of the point-spread-function in the ${\mathcal {A}}$-space. The work is inspired by previous results in OPT, where the auto-correlation is used to perform alignment-free reconstructions^11^. The use of ${\mathcal {A}}$ was possible because it commutes with the projection operator^12^. Here, instead, we calculate a tomographic auto-correlation of the sample based on multi-view light-sheet acquisitions. Fusing them leaves us with an ensembled ${\mathcal {A}}$, created without aligning the views. It constitutes our starting point for the reconstruction: by inverting ${\mathcal {A}}$, we form a tomographic view aligned at the sub-pixel level. Furthermore, we demonstrate that this inversion turns into a reconstruction sharper than the average fusion carried out in direct space. Since it is desirable to take into account the resolution-loss determined by the finite aperture of the optical system^13^, our protocol can further accomplish simultaneous deconvolution with a modified Bayesian ${\mathcal {A}}$ inversion scheme. For this study, the use of powerful GPUs plays a crucial role due to the computational complexity of our protocols. Without graphics cards, the reconstruction problem presented here would remain just a mere theoretical exercise.

**Figure 1:**
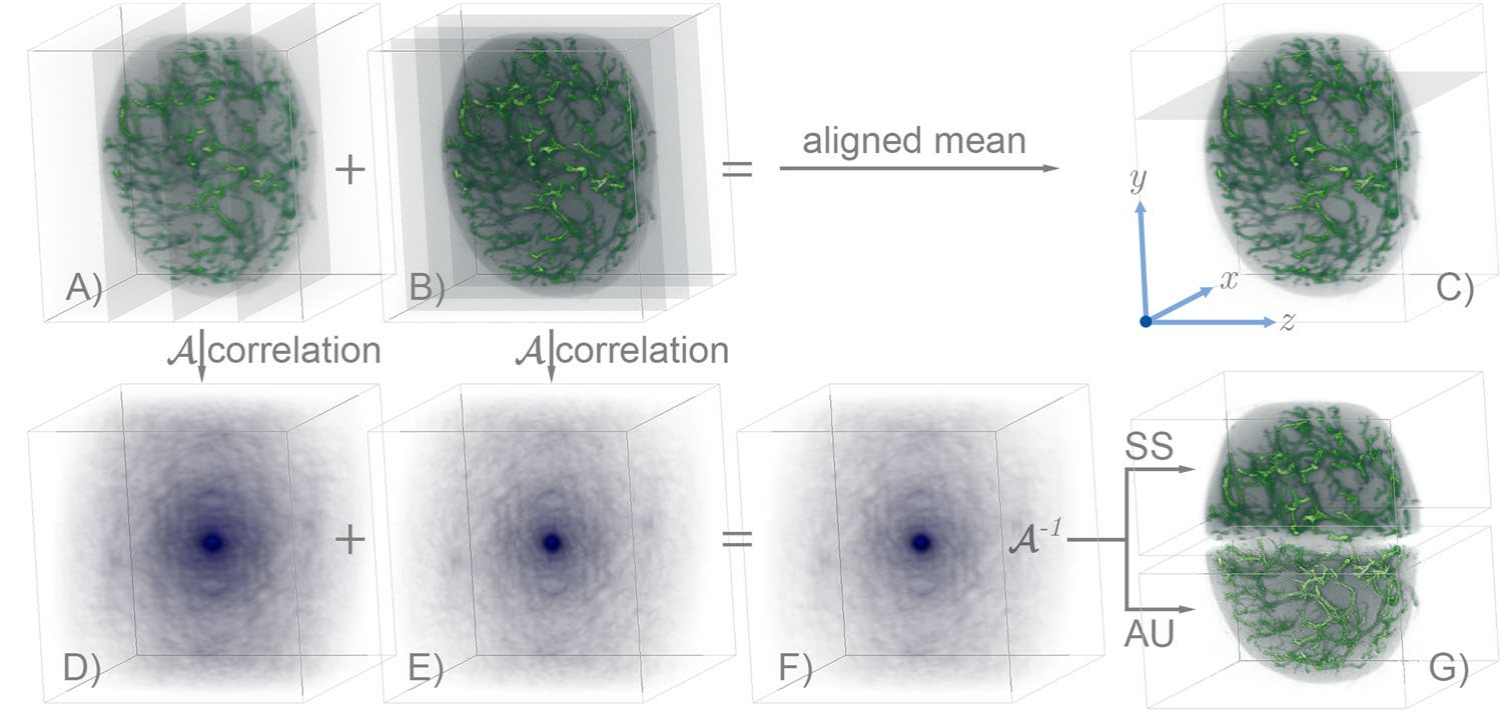
Reconstruction pipeline. (**A**) Rendering of the reference view taken at $\varphi = 0^\circ$. The planes indicate the *xy*-camera acquisition along the *z*-scanning direction. (**B**) Orthogonal detection by rotating the sample at $\varphi = 90^\circ$. (**C**) Aligned-average of 12 measurements. The axes are chosen according to the reference view (*x*-lateral, *y*-transverse, *z*-longitudinal). (**D**) Auto-correlation of the view at $0^\circ$. (**E**) ${\mathcal {A}}$ of the view at $90^\circ$. (**F**) ${\mathcal {A}}$ averaged through 12 angles. (**G**) Reconstructions obtained by using deauto-correlation methods. For visual comparison, the upper part shows the result using the Schultz-Snyder protocol. The bottom one compares it with that of the Anchor-Update method.

## Results

Our reconstruction strategy is grounded on the property of the auto-correlation of being centered in the shift-space. Each observation of the object is auto-correlated, and it concurs at the formation of the tomographic average ${\mathcal {A}}$ of the sample. Let us use the subscript $\mu$ to indicate the stack obtained by camera acquisitions and the superscript $\varphi _i$ to denote its angular orientation indexed by *i*. In an experimental measurement, we observe a blurred version of the object due to the point spread function (PSF) of the system *h*, further corrupted by the presence of the noise $\varepsilon$. A typical acquisition is rendered in Fig. 1A, where we display a volumetric object imaged with a light-sheet microscope at the reference angle of $0^\circ$. For the moment, we use Fig. 1 just for the discussion of the reconstruction pipeline; we will present the details about the specimen and the setup afterward. We assume that the additive $\varepsilon$ can be neglected in case of high signal-to-noise ratio measurements. Now, we arrange the auto-correlation in a more convenient form. By applying the operator ${\mathcal {A}}$ to a given stack (see the Methods), we have that:

1 $$\begin{aligned} \chi _\mu \equiv&{\mathcal {A}}\{o_\mu \} = o_\mu \star o_\mu = \left( o*h \right) \star \left( o*h \right) \end{aligned}$$

2 $$\begin{aligned} =&\chi * {\mathcal {H}} = o * {\mathcal {K}} . \end{aligned}$$

here, $\chi =o\star o$ is the ideal auto-correlation of the object, ${\mathcal {H}}={\mathcal {A}}\{h\}$ is the PSF in auto-correlation space and ${\mathcal {K}} = o \star {\mathcal {H}}$ is an effective kernel. Figure 1D shows the auto-correlation of the volume displayed in panel A. The first equality in Eq. (2) implies that the auto-correlation of the ideal object is blurred by ${\mathcal {H}}$, given by the auto-correlation of the direct space PSF *h*. The second indicates that $\chi _\mu$ can be seen as a convolution of the object with a blurring kernel that contains the object itself. We consider *N* evenly rotated measurements that we denote with the index $\varphi _i$. The rotation of each measurement back to the reference angle $0^\circ$ by $-\varphi _i$ is the only pre-processing step required. Additionally, we subtract the mean value of a dark region where the sample is not present. In Fig. 1B, we display an orthogonal acquisition which was rotated by $90^\circ$ to match the angular view of Fig. 1A, and then used to compute the auto-correlation (Fig. 1E). Denoting each observation as $o^\varphi _\mu$, and its corresponding ${\mathcal {A}}$ as $\chi ^\varphi _\mu$, the quantities of interest are the averages:

3 $$\begin{aligned} \overline{o}_\mu = \frac{1}{N} \sum _{i=0}^{N} o_\mu ^{\varphi _i}, \quad \overline{\chi }_\mu = \frac{1}{N} \sum _{i=0}^{N} \chi _\mu ^{\varphi _i} \quad {\text {and}} \quad \overline{\mathcal {H}} = \frac{1}{N} \sum _{i=0}^{N} {\mathcal {H}}^{\varphi _i}. \end{aligned}$$

Before computing the fusion as $\overline{o}_\mu$ (that we consider as the standard reconstruction, rendered in Fig. 1C), each measurement required an accurate alignment against the reference. The $\overline{\chi }_\mu$ displayed in Fig. 1F, instead, is accurate because the auto-correlations are centered by definition. Ideally, this implies that we can obtain an intrinsically aligned average-reconstruction^14^ from $\overline{\chi }_\mu$, provided that we have a robust way to carry out the inversion $\overline{o}_\rho = {\mathcal {A}}^{-1}\{\overline{\chi }_\mu \}$. The rigid shifts between different observations are encoded in their Fourier phase, which we always discard when working in the ${\mathcal {A}}$-space. Instead, we retain only the information coming from the Fourier transformations of each acquisition. By inverting $\overline{\chi }_\mu$, we implicitly look for a new phase of the object that represents an overall alignment between each of the views. In fact, this problem falls within the class of phase retrieval (PR) since we have access to the Fourier modulus of a real object, but the phase information is missing^15^.

**Figure 2:**
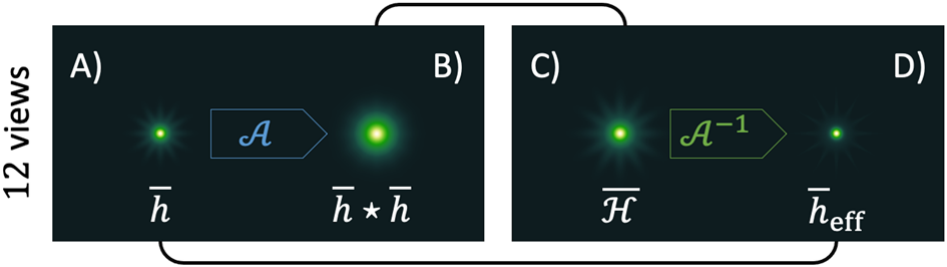
Point-spread-functions analysis. (**A**) PSF that blurs $\overline{o}_\mu$. (**B**) Auto-correlation of $\overline{h}$. (**C**) PSF $\overline{\mathcal {H}}$ that blurs the average $\overline{\chi }_\mu$. (**D**) Corresponding PSF in the object domain, sharper than $\overline{h}$.

**Figure 3:**
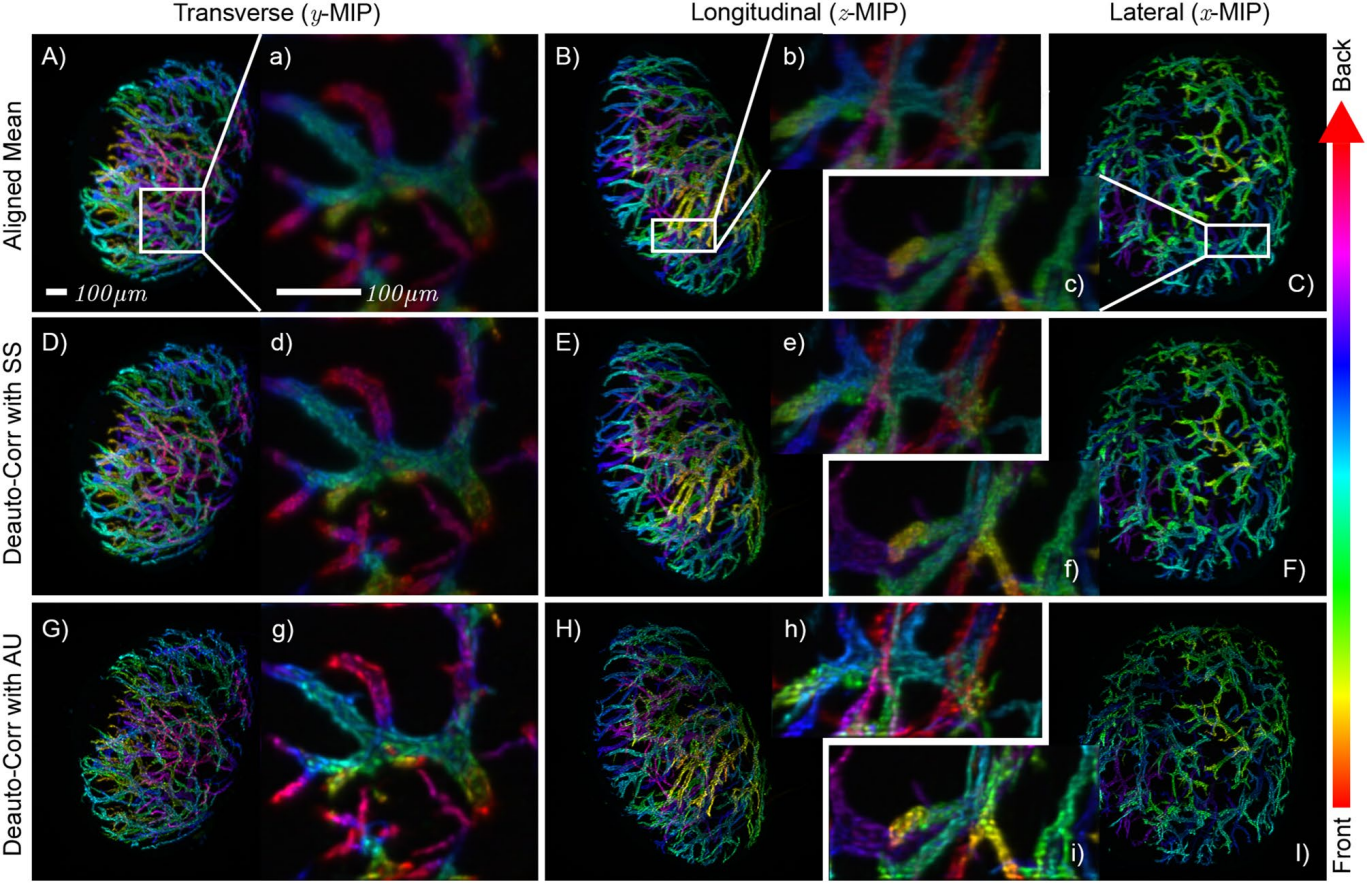
Methods comparison for the reconstruction of the vasculature in a mouse popliteal lymph node. The quality of the first row improves in the central and in the bottom rows. Results of aligned mean shown in a transverse (**A**), longitudinal (**B**) and lateral (**C**) direction. The shown data are color-encoded maximum intensity projection (MIP)^16^ along each spatial coordinate. In all these MIPs, the color indicates the depth at which the corresponding feature is located. The small letters indicate the cropped volume. The cropped regions located within the whole specimen are framed with white boxes. The scale bar is $100 \mu m$. (**A**, **a**) Transverse view of the volume $\overline{o}_\mu$ averaged and aligned by cross-correlation (Rendered in Fig.1, viewed from the top). (**B**, **b**) Longitudinal (or side) view projection. (**C**, **c**) Lateral (or front) view. (**D**, **d**) Transverse, (**E**, **e**) longitudinal, and (**F**, **f**) lateral projections of the volume $\overline{o}_\rho$ reconstructed with SS. (**G**, **g**) Transverse, (**H**, **h**) longitudinal, and (**I**, **i**) lateral projections of the volume $\overline{o}$ deconvolved with AU.

With these two quantities in hand, we try to extrapolate intrinsically-aligned reconstructions by inverting the ${\mathcal {A}}$ with two schemes:

(I). Given $$\overline{\chi }_\mu = \overline{o}_\rho \star \overline{o}_\rho$$, find $$\overline{o}_\rho$$;
(II). Given $\overline{\chi }_\mu = \left( \overline{o} \star \overline{o} \right) * \overline{\mathcal {H}}$, deblur it by $\overline{\mathcal {H}}$ and find $\overline{o}$.

At first sight, only the second scheme that deconvolves the PSF appears to provide a super-resolved reconstruction. However, the scheme (I) implies something even more interesting that we describe in Fig. 2. By averaging $o_\mu ^{\varphi _i}$ in direct space, the resulting volume gets blurred by an average PSF given by $\overline{h} = \frac {1}{N} \sum\nolimits _i h^{\varphi _i}$ (Fig. 2A). Its ${\mathcal {A}}\{\overline{h}\}=\overline{h}\star \overline{h}$ can be visualized in Fig. 2B. By averaging auto-correlations, instead, we neglect second-order cross-terms of the PSF. Those contributions introduce long-correlations in the fused image and degrade the image quality. For comparison, the corresponding ${\mathcal {A}}$-PSF is shown in Fig. 2C. As a consequence, by solving for $\overline{o}_\rho$, we achieve an effective PSF that is sharper than $\overline{h}$. Interestingly, this is an implicit property that comes along with the average of multiple views of ${\mathcal {A}}$. Thus, the resolution gain is attainable without having access to the PSF of the system. However, for comparison, we show the effective point-spread function achieved $\overline{h}_{\text {eff}}={\mathcal {A}}^{-1}\{\overline{\mathcal {H}}\}$ in Fig. 2D. For a detailed discussion, see the Supplement Materials. We decided to tackle the scheme (I) by using the Schultz-Snyder (SS) iterations^17^:

4 $$\begin{aligned} o^{t+1} = o^{t} \left[ \left( \frac{\chi _\mu }{o^{t} \star o^{t}}\right) *\widetilde{o}^{t} + \left( \frac{\chi _\mu }{o^{t} \star o^{t}}\right) \star o^{t} \right] . \end{aligned}$$

For the scheme (II), instead, we implement the Anchor-Update (AU) protocol^18^ that was developed ad-hoc for this purpose:

5 $$\begin{aligned} o^{t+1} = o^{t} \left[ \left( \frac{\chi _\mu }{o^{t} * {\mathcal {K}}^{t}}\right) *\widetilde{\mathcal {K}}^{t}\right] , \quad {\text { updating: }} {\mathcal {K}}^{t} = o^t \star {\mathcal {H}}. \end{aligned}$$

Both are fixed-point iterative Bayesian methods, having the number of iterations as the only parameter to set. In the present case, we set a high number of $5 \cdot 10^5$ iterations for both since these methods are very stable and can withstand long runs. On the other hand, this is also a drawback since these algorithms suffer from a slow convergence rate (each update $t+1$ is close to the previous one *t*).

To delve into the proposed method, let us consider volumetric acquisitions taken with an LSFM setup of a cleared mouse popliteal lymph node^19^. We are interested in reconstructing the three-dimensional vasculature stained with a fluorescent label. The stack $o_\mu ^\varphi$ constitutes a single volumetric view of the specimen and contains the camera detections of the sample scanned through the light sheet. We use 12 volumes by rotating the sample in steps of $30^\circ$. The $o_\mu ^\varphi$ acquired at $0^\circ$ and $90^\circ$ were already rendered in Fig. 1A, B. Standard multi-view reconstruction algorithms require the alignment of every dataset against the reference view (that we assume at $\varphi = 0^\circ$). A consolidated strategy (accurate at pixel level) is to locate the maximum of the cross-correlation between the reference and the view, translating it back accordingly. However, the researcher may be looking for sub-pixel accuracy, which would require to upsample the volume accordingly to the resolution that he wants to reach^20^. This condition makes the size of the problem rapidly explode, leaving the user with an up-sampled estimation (compared to the original measurement) that needs to be down-sampled for the formation of the final image (Supplement Materials). Here, instead, we produce a multi-view reconstruction that is accurate at the sub-pixel level and directly formed at the original resolution. We do not calculate any volume translation; we simply process the reconstruction altogether starting from its auto-correlation $\overline{\chi }_\mu$.

**Figure 4:**
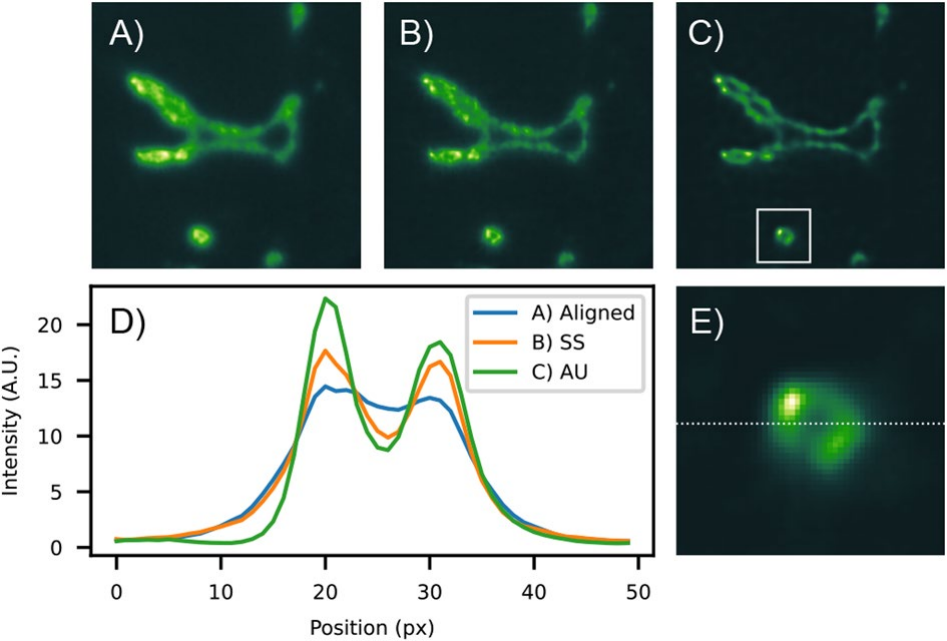
Tomographic slice of the cropped volume. (**A**) Aligned mean (standard reconstruction). (**B**) Reconstruction using SS. (**C**) Reconstruction using AU. (**D**) Profile plot along the dashed line in panel (**E**) for the three cases. (**E**) Detail of the small opening for AU.

We analyze two experimental situations. In Figs. 3 and 4, we report the results obtained on two regions of the specimen. The first contains the whole specimen and corresponds to a volume of $512^3$ voxels, with a size of (1320 μm)^3^. The second volume takes a region of interest of $256 \times 256 \times 128$ voxels, with a size of 330 μm × 330 μm × 165 μm. Convolutions and correlations are implemented via Fast-Fourier Transform (FFT) spectral decomposition. The GPU implementation is essential to perform such reconstructions since the method relies on intensive usage of 3D-FFT. We implemented the code in Python by using the CuPy library^21^, which provides a flexible CUDA framework for matrix operations. The problems were tackled using a single Nvidia Titan RTX, equipped with 4608 CUDA cores and 24 gigabytes of RAM. Each step is accomplished in 0.48 s for the first volume, while the second needs 0.05 s. We choose the reference view at angle $\varphi =0^\circ$ for the initial guess at $t=0$. The results obtained for the reconstructions of the whole specimen are rendered in Fig. 1G, where the top-half is the result of SS, and the bottom half is the result of AU. We show the maximum intensity projection (MIP) along each spatial coordinate to compare the different results. The top row of Fig. 3 shows the ground-truth reconstruction, obtained by averaging the views previously aligned by locating the peak of their cross-correlation. The second row of Fig. 3, instead, shows the results of SS iterations. Since the reconstruction is formed from an inherently aligned auto-correlation, the features of the specimen are better resolved than the standard reconstruction. Compared to the $\overline{o}_\mu$, the reconstructed $\overline{o}_\rho$ is crisp, with sharp features better isolated from an intensity background. The reconstruction contrast improved due to the sharper PSF $\overline{h}_{\text {eff}}$ implied by the usage of the auto-correlation. The third row of Fig. 3 displays the results obtained with AU, deconvolving ${\mathcal {H}}$ from the estimated auto-correlation. The final effect is a deblurring of the reconstruction with respect to the SS. We can further assess the effectiveness of the method by examining a tomographic slice taken through the middle of the full-resolution volume. In Fig. 4A, we show the standard reconstruction result of the aligned and averaged volume. We have chosen a detailed region that displays a bifurcated blood vessel and a smaller circular opening located at the bottom. Figure 4B slices the same plane of the volume $\overline{o}_\rho$ after the inversion of $\overline{\chi }_\mu$ via SS iterations. If we compare it with the standard result, we observe a clear improvement in the reconstruction quality. Having correctly reinterpreted sub-pixel misalignment and with a neat PSF, the image is rich in details and well contrasted, where the standard reconstruction appears fuzzier. On the other hand, Fig. 4C shows the reconstruction of the same volume by using AU. Here, it is possible to appreciate the deblurring effect that leaves us with a highly-resolved reconstruction. To assess the qualitative verdict of our analysis, we examine a small detail of the vessel located at the bottom of panel C. The region of interest is displayed in panel E as a reference in the case of AU reconstruction. We draw a line profile in the middle of it, and we plot the intensities for each of the reconstructions considered in Fig. 4D. The standard reconstruction almost confuses the walls of the small blood vessel, whereas SS resolves this detail. The opening within the blood vessel becomes even more evident when we use AU, given that the simultaneous PSF deconvolution lets us resolve sharper details. Thorough image analyses are presented in the Supplement Materials document accompanying this manuscript.

## Discussion

It is worth stressing that our auto-correlation method goes beyond the deconvolution approach. We are exploring a new path in alignment-free image formation, studying its advantage in terms of PSF. With this work, we presented an approach to the problem of shift-invariant reconstructions in volumetric multi-view tomography. Rather than relying on alignment and fusion pipelines, we proposed a conceptually simple approach that promotes the reconstruction into the shift-invariant ${\mathcal {A}}$-space. We made use of multiple views of the specimen with the sole goal of refining the estimation of the auto-correlation of the object since we consider it as the ideal quantity for the formation of inherently aligned reconstructions. Since the user is free from the alignment task, one could direct his attention to better ways to estimate the auto-correlation. In particular, this may open the path for the corrections of higher-order transformations as, for example, those introduced by inaccuracies of the rotation stage. Two-axes angular tilts are seen easier in the shift-invariant space than in the object space since we no longer worry about the object positioning. Furthermore, we have proven that the solution of the ${\mathcal {A}}^{-1}$ can be accompanied with deconvolution^18^. Concatenating two inverse problems can be hazardous since remaining artifacts from the first inversion may condition the behavior of the following method. Technically, these problems can only converge if we pad enough the reconstruction volume: the auto-correlation of a discrete *n*-signal is defined on a translation-space that is $2n-1$ long. However, we found that starting with a close guess ensures convergence even for volumes that do not comply with appropriate frequency padding. In volumetric tomography, this guess may be either a single view or the aligned mean of the specimen since both are not distant from the ideal reconstruction. Unpadded volumes are smaller, and this lets us save computer memory and speeds up the execution. The main computational burden of our algorithms, however, is performing convolutions of large volumes. Processing $10^5$ SS-iterations on a volume of $512^3$ voxels takes about 80 min. Additionally, each AU step needs one convolution more than SS, and it is typically 25% slower. Novel GPU architectures continuously speed up those operations, and there are several ways to implement convolutions (direct, Fourier, or overlap-add methods) optimized for the size of the problem considered. In any case, both SS and AU are Bayesian quadratic methods and typically require many iterations to converge^17,22^. With this respect, a new approach to Bayesian deconvolution^23^ managed to reduce by two orders of magnitude the iterations needed by tuning the forward and backward projection operators. Those operators are similar to what we have -respectively- at the denominator and numerator in our Eq. (5) and, in the future, we may consider the adoption of a similar approach to increase efficiency. Both the iteration number and time consumption are crucial aspects that we plan to investigate further, and that may decrease the processing time from a few hours to a fraction of it.

## Methods

### Image pre-processing

Each raw dataset was subtracted with a corresponding average background value, rotated to the same angular orientation of the first dataset acquired at $\varphi =0$. The PSF of the system was assumed to be Gaussian, elongated along the direction of scanning. For each of these stacks, we computed the corresponding auto-correlation sequence. We took the absolute value of the average auto-correlation to avoid any presence of unwanted negative values. These are determined by the background-subtraction and eventually by rounding errors due to FFT computation.

### Multi-view registration and fusion

As standard reconstruction comparison, we aligned the views against each other by finding the location of the maximum of the cross-correlation between ${\mathcal {X}}\{o_\mu ^i;o_\mu ^j\}$, for $i \ne j$. We defined the displacement vector $\pmb {m}_i$ with respect the central coordinate $\pmb {\xi }=\pmb {0}$. We kept $\varphi _{0}=0^\circ$ as a reference and we translated each $\varphi _i$ by the vector $-\pmb {m}_i$ defined in this way. Then we computed the average of the registered stacks to form $\overline{o}_\mu$.

### Rearranging the auto-correlation

We suppose that a generic measurement $o_\mu$ is described by:

6 $$\begin{aligned} o_\mu = o * h + \varepsilon . \end{aligned}$$

We neglect the additive noise by assuming $\varepsilon =0$. Using the commutation properties of the convolution-correlation, the relations $\left( a * b \right) \star \left( a * b \right) = \left( a \star a \right) * \left( b \star b \right)$ and $a \star \left( b*c\right) =\left( a \star b\right) *c$, we have that:

7 $$\begin{aligned} \chi _\mu \equiv&{\mathcal {A}}\{o_\mu \} = o_\mu \star o_\mu = \left( o*h \right) \star \left( o*h \right) \end{aligned}$$

8 $$\begin{aligned} =&\left( o\star o \right) * \left( h\star h \right) = o * \left( o \star \left( h\star h \right) \right) \end{aligned}$$

9 $$\begin{aligned} =&\chi * {\mathcal {H}} = o * {\mathcal {K}}. \end{aligned}$$

here we have called $\chi =o\star o$, ${\mathcal {H}}=h\star h$ and ${\mathcal {K}}=o \star {\mathcal {H}}$. In the body of the article, we use only the last two equations.

### Experimental details

For the tests performed in our study, we use the image data taken from the work of Ozga et al.^19^, to which we refer for the experimental protocols. The sample, provided by Prof. J. Stein and imaged by J. Swoger, is a cleared mouse popliteal lymph node having the vasculature stained with the Alexa Fluor 488 dye (HEV, high endothelial venule). This specimen was embedded in agarose, then cleared and imaged in Benzyl-Alcohol Benzyl-Benzoate (BABB). The fluorescence was excited with a light-sheet perpendicular to the camera detection at $\lambda _{exc} = 488$ nm, imaged onto the sample with a 2.5×/0.07 N PLAN (air) objective lens. With a band-pass filter at 525/50 nm, they recorded the emitted fluorescence using a 5×/0.12 N Plan EPI (air) objective lens. The sample was scanned through the light sheet along the z-axis, perpendicularly to the camera detection in steps of 4.985 μm.

## Supplementary Information

Supplementary Information.
