# Supplementary material for: Beyond multi view deconvolution for inherently aligned fluorescence tomography
Source: Sci Rep. 2021 Aug 3;11:15723. doi: 10.1038/s41598-021-95266-2 (PMC8333050; doi:10.1038/s41598-021-95266-2)
Supplement: Supplementary file 1 — Supplementary Information. [file 41598_2021_95266_MOESM1_ESM.pdf]

Beyond multi view deconvolution for inherently aligned fluorescence tomography: Supplementary Material

Daniele Ancora[1,*], Gianluca Valentini[1,2], Antonio Pifferi[1,2], and Andrea Bassi[1,2]

[1]Dipartimento di Fisica, Politecnico di Milano, Piazza Leonardo da Vinci 32, 20133 Milano, Italy
[2]Istituto di Fotonica e Nanotecnologie, Consiglio Nazionale delle ricerche, Piazza Leonardo da Vinci 32, 20133 Milano, Italy
[*]corresponding author: daniele.ancora@polimi.it

ABSTRACT

The present supplement document accompanies the main article and describes, in detail, the materials and methods used to replicate the results. Here, we analyze the intrinsic sub-pixel alignment provided by the reconstruction from auto-correlations. Furthermore, we include the considerations concerning the multi-view approach and a thorough study on the PSF implied by inverting an average auto-correlation.

1 Correlation/Convolution Theory

In the following section, we define the quantities used in the manuscript. We make use of the bold $\boldsymbol{x} = (x, y, z)$ to indicate a vector in the direct space $\boldsymbol{x} \in \mathbb{R}^3$. Reconstructions are always finalized in the direct $\boldsymbol{x}$-space. Correlations and convolutions are quantities defined by relative shifts in $\mathbb{R}^3$, which we define with the vector $\boldsymbol{\xi} = (\xi, \eta, \zeta)$. Also $\boldsymbol{\xi} \in \mathbb{R}^3$ and we refer to as the shift-space. We recall that the Fourier transform $\mathcal{F}$ of a function $h(\boldsymbol{x})$ is defined as:

$$\mathcal{F}\{h\} \equiv \int_{-\infty}^{\infty} h(\boldsymbol{x})\, e^{-i2\pi \boldsymbol{x}\cdot\boldsymbol{k}}\, d\boldsymbol{x} = \hat{h}(\boldsymbol{k}), \tag{1}$$

where $\boldsymbol{k} = (k_x, k_y, k_z)$ is the wave-vector in frequency space. Conceptually, the cross-correlation is defined as a reference function o multiplied by another function h that shifts by $\boldsymbol{\xi}$ as in:

$$o \star h = \mathcal{X}\{o; h\} = \int \overline{o(\boldsymbol{x})} h(\boldsymbol{\xi} + \boldsymbol{x})\, d\boldsymbol{x}. \tag{2}$$

The cross-correlation theorem states that $o \star f$ can be written as a product in the Fourier domain:

$$\mathcal{F}\{o \star h\} = \overline{\mathcal{F}\{o\}} \cdot \mathcal{F}\{h\}. \tag{3}$$

For the implementation of our method, we make intensive use of this property and the following Fourier theorems. The auto-correlation is defined as a quantity shifted and multiplied by itself as in:

$$o \star o = \mathcal{A}\{o\} = \int \overline{o(\boldsymbol{x})} o(\boldsymbol{\xi} + \boldsymbol{x})\, d\boldsymbol{x} \equiv \chi(\boldsymbol{\xi}). \tag{4}$$

The Wiener-Khinchin (power-spectrum) theorem states:

$$\mathcal{F}\{o \star o\} = \|\mathcal{F}\{o\}\|^2. \tag{5}$$

The auto-correlation of any real signal is an even function, thus $\chi(\boldsymbol{\xi}) = \chi(-\boldsymbol{\xi})$, and it is always centered around its maximum located at $\boldsymbol{\xi} = (0, 0, 0) \equiv \mathbf{0}$. The convolution is defined by shifting and multiplying two functions o and h as in:

$$o * h = \mathcal{C}\{o; h\} = \int o(\boldsymbol{x})\, h(\boldsymbol{\xi} - \boldsymbol{x})\, d\boldsymbol{x}. \tag{6}$$

The convolution theorem states that:

$$\mathcal{F}\{o * h\} = \mathcal{F}\{o\} \cdot \mathcal{F}\{h\}. \tag{7}$$

2 The point-spread-function of the auto-correlation in the object space

One of the most relevant aspects of this method is its intrinsically sharper point-spread function, determined by the fusion of the auto-correlation. By using only auto-correlations, we discard background components in direct space linked to cross-correlation terms. The scheme in Fig. 1 exemplifies this by numerically examining this aspect. Let us suppose that we want to fuse two orthogonal views; the PSF of the fused reconstruction is a cross elongated towards both directions of scanning. Now we examine how this average is viewed in the auto-correlation space. We call as h^{0° and h^{90° the PSFs of the orthogonal views that we fuse. In auto-correlation space, we have that:

$$\overline{h} \star \overline{h} = \left(h^{0^\circ} + h^{90^\circ}\right) \star \left(h^{0^\circ} + h^{90^\circ}\right) \tag{8}$$

$$= h^0 \star h^0 + h^0 \star h^{90} + h^{90} \star h^0 + h^{90} \star h^{90} \tag{9}$$

$$= \mathcal{H}^0 + \mathcal{H}^0_{90} + \mathcal{H}^{90}_0 + \mathcal{H}^{90} = \mathcal{H}_{\text{tot}}. \tag{10}$$

By taking the average auto-correlation, instead, we are selecting only two of the 4 terms in Eq. 10. This implies that the following condition is satisfied:

$$\mathcal{H}_{tot} > \mathcal{H}_0 + \mathcal{H}_{90} \equiv \overline{\mathcal{H}}, \tag{11}$$

which holds also when we return to the object domain:

$$\overline{h} > \overline{h}_{\text{eff}}. \tag{12}$$

In the equation above, $\overline{h}_{\text{eff}} = \mathcal{A}^{-1}\{\overline{\mathcal{H}}\}$ is defined as the quantity which gives rise to $\overline{\mathcal{H}} = \overline{h}_{\text{eff}} \star \overline{h}_{\text{eff}}$. A numerical example is presented in Fig. 1, where we use a two-dimensional Gaussian PSF having for the detection side a $\sigma_{det} = 0.90\mu m$ and along the scanning axis, $\sigma_{scan} = 4.32\mu m$. These are the values from a Gaussian fit of the parameter given by the experimental detection considered.

Fig. 1 follows exactly the scheme of Eqs. 8-10. We notice how the cross-correlation terms present in the average PSF contribute as a background over the average auto-correlation PSF $\overline{\mathcal{H}}$ that we take advantage of. By inverting the auto-correlation then, we converge to a reconstruction free from any translational misalignments and, also, we achieve a sharper image since the underlined PSF is smaller than the original one.

2.1 PSF limit for an infinite number of observations

We have seen how working with the average auto-correlation gives, in turn, a sharper PSF for the reconstruction of $\overline{o}_\rho$ in the direct space. Explicitly, this was proven theoretically for two views, whereas, here we study what happens when the number of measurements tends to infinity. To reproduce infinite angular views, we rotate the PSF by steps of $1°$, and we calculate its average over 360 equally spaced angular rotations.

The results are presented in Fig. 2. The top row reassembles the results obtained when fusing two views and the bottom row compares it with its limit reached for an infinite number of observations. We notice once again how $\overline{h}_{\text{eff}}$ is always sharper than its counterpart in the direct space $\overline{h}$.

3 Sub-pixel accuracy in multi-view deauto-correlation based on Schultz-Snyder

3.1 Numerical study

It is necessary to verify the validity of the reconstruction pipeline and the effect given by using auto-correlation instead of direct object observations. In our article, the predominant effect determined by inverting the averaged auto-correlation is its intrinsic sharper PSF. It is hard to highlight a sub-pixel error when such a macroscopic effect takes place simultaneously. To convince the reader of the intrinsic sub-pixel alignment capability, we run an ad-hoc numerical experiment inspired by an analogous study on sub-pixel registration algorithms[1]. We generate a bi-dimensional $512^2 px$ sample that mimics the vascular net of the specimen examined in the main article. We work on a slice to ease the comparison. In three dimensions, the alignment effect will also compensate for the displacements along the third direction. We proceed as follows. To begin with, we define the ground truth measurement, averaging

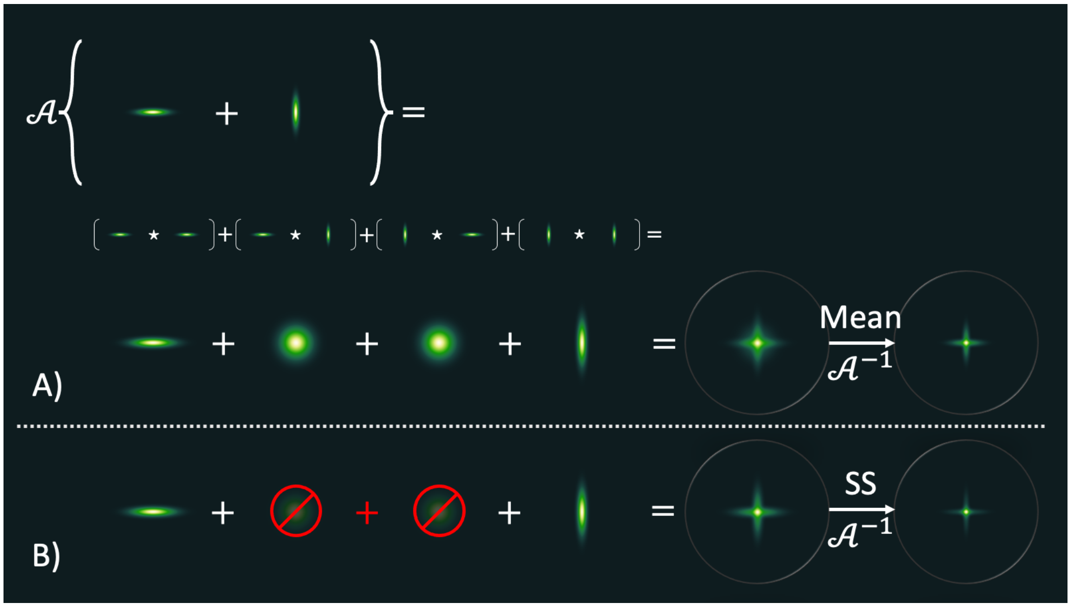

Figure 1. PSF math in auto-correlation space. A) Visual representation of the results of Eqs. 8-10. B) Visual representation of Eq. 11. With the red symbols we indicate the background contributions excluded when averaging the $\mathcal{A}$.

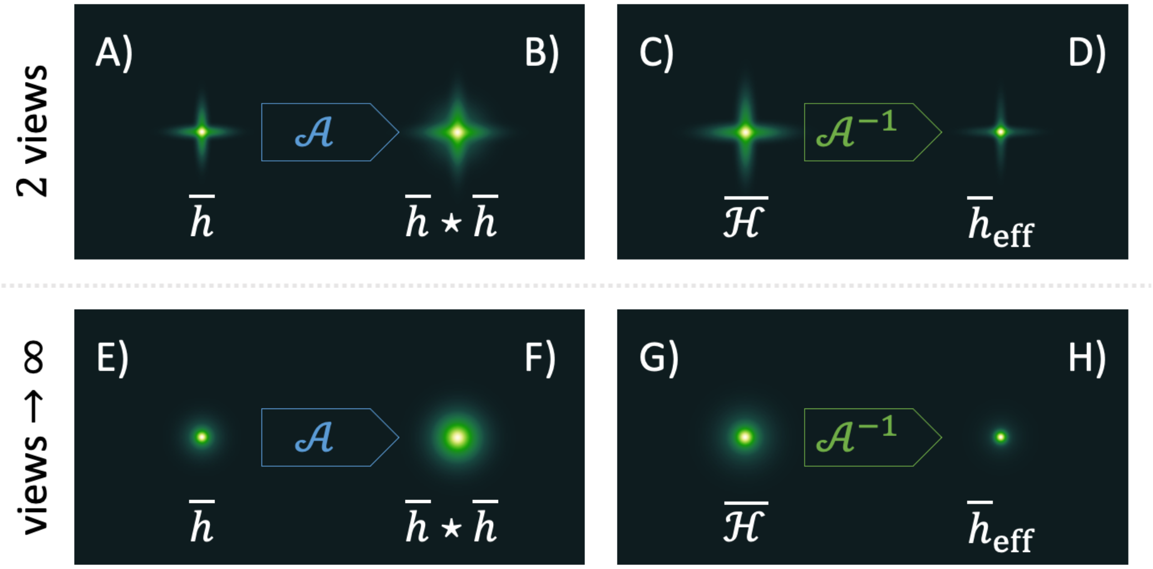

Figure 2. PSF analysis when the number of measurements $N \to \infty$. A) Average of orthogonal PSFs in direct space. B) Auto-correlation of the average PSF. C) Average auto-correlation PSF. D) Corresponding effective PSF in direct space given by the summation of the $\mathcal{A}$. E) PSF in direct space with ∞ measurements. F) Its auto-correlation. G) Average auto-correlation PSF for $N \to \infty$. H) The $\overline{h}_{\text{eff}} = \mathcal{A}^{-1}\{\overline{\mathcal{H}}\}$.

8 times the same object with added Poisson noise with $\lambda = 0.05$. This estimation, reported in Fig. 3a, is not affected by any misalignment; it just averages over different noisy realizations. Then, we upsample the generated slice by 10 times, obtaining an image size of $5120^2 px$, and we add a 2D random shift $\in [-10, 10]\, px$ which corresponds to a less-than-a-pixel-shift within the original image. We repeat this procedure for eight measurements. After, we downsample these -slightly- shifted images to reach the original measurement size, and then we add the noise, as in the previous case. Taking the average of this dataset forms an estimation of the original image affected by a

sub-pixel shift. We show the result of this procedure in Fig. 3b. Now we implement the method that we proposed in the manuscript: rather than averaging the sub-misaligned images in the direct space, we take the average of the auto-correlations (Fig. 3d). Since we did not include any directional blurring, there are no cross-correlation terms neglected that contribute to the final PSF of the image. The averaged auto-correlation is, then, inverted using 10^6 iterations of the Schultz-Snyder algorithm. The resulting image in Fig. 3e is a sharp reconstruction of the object. To compare the images, we take inspiration from the article[1] that analyzes sub-pixel registration procedures. The metric used to evaluate the reconstruction quality is the normalized root-mean-square error (NRMSE) between two images $f(x, y)$ and $g(x, y)$:

$$E^2 = 1 - \frac{\max |f(x, y) \star g(x, y)|^2}{\sum_{x,y} |f(x, y)|^2 \sum_{x,y} |g(x, y)|^2}. \tag{13}$$

By definition, NRMSE is invariant to image shifts, and a low value corresponds to better reconstructions. According to NRMSE, the average reconstruction affected by sub-pixel misalignment turns to an $E = 0.184$, while the reconstruction obtained from auto-correlations reaches a lower value, $E = 0.070$. The image reconstructed with SS is closer to the original one. To further verify this, we align both reconstructions by locating the peak of their cross-correlation against the ground truth. Then, we calculate the euclidean distance between the ground truth and the reconstructions. Also, in this case, the misaligned reconstruction distance $d = 12.47$ turns to be higher than the reconstruction based on auto-correlation, $d = 4.76$. It is possible to assess this discrepancy also by looking at the absolute difference between the ground truth against the sub-misaligned image (Fig. 3c) and the SS-reconstruction (Fig. 3f). Since the only perturbation included in this numerical experiment was a sub-pixel misalignment, we can conclude that inverting the average auto-correlation also compensates for this effect.

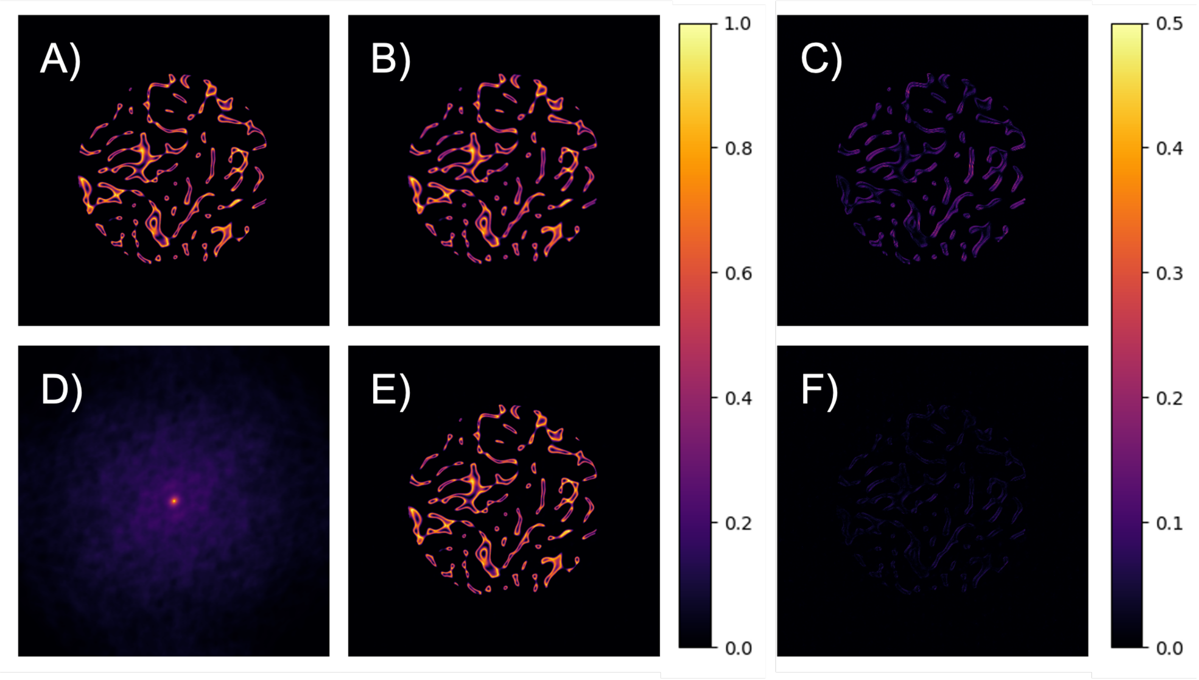

Figure 3. Numerical study to assess sub-pixel intrinsic alignment. A) Ground truth image averaged over 8 aligned noisy acquisitions. B) Reconstruction affected by sub-pixel misalignment. C) Absolute difference between the ground truth and misaligned reconstruction. D) Average auto-correlation of sub-misaligned data. E) Reconstruction based on SS auto-correlation inversion. F) Absolute difference between ground truth and SS reconstruction. The darker image implies a reconstruction that is closer to the actual object than the sub-misaligned image.

3.2 Experimental verification

Here, we consider the slices taken from the cropped volumes at full resolution, previously aligned. We fuse them by taking their average to form the ground truth. Consequently, we proceed as follows. We upsample each view by a factor of 10, padding the Fourier transform appropriately (thus, a single slice becomes 2560×2560). By doing

this, each pixel in the new image is 1/10 of the previous size. With this size increase, we can correct discrete displacements with an accuracy of a fraction of the original image resolution. At this upsampling factor, the volumetric reconstruction of the new volume would become very demanding in terms of memory usage. We decide to work on a two-dimensional slice because, currently, there is no hardware able to perform FFT directly on volumes of 2048^3 voxels. We align each upsampled view against the reference; this is the reconstruction corrected for the misalignment of a tenth of a pixel.

Instead, we use SS[2] to invert the auto-correlation averaged as explained in the main article. Before comparing the results with the SS reconstruction, let us recall that inverting the average of the auto-correlation leaves us with a more compact PSF, as described in the Sec. 2. Although this is an important result, we isolate the effect of sub-pixel accuracy from the effect on the PSF. To do this, we have to take all the terms of the cross-correlations, not only the auto-correlations. The terms that we include in the summation are:

$$\overline{\mathcal{C}}_\mu = \sum_{i,j=0}^{N} \mathcal{X}\{o_\mu^{\varphi_i}; o_\mu^{\varphi_j}\} \tag{14}$$

for all the possible combinations of i and j. With this, we form an average cross-correlation that we invert by using SS. Please note that taking the auto-correlation reduces this summation only to the terms with $i = j$.

Since we are also interested in testing the effect of long runs, we complete two sets of 1 million of iterations for the reconstructions starting from the cross- and auto-correlation averages. By analyzing the results in Fig. 4, it appears clear that upsampling the slice and aligning it (panel B) does not improve much the reconstruction quality against the aligned mean (panel A). We point out that by running SS on the cross-correlation mean $\overline{\mathcal{C}}_\mu$ leads to the reconstruction of the slice (panel C) that closely matches the upsampled one in panel B. We can observe it also by looking at the profiles on the plot underneath. The upsampled reconstruction and the one obtained by inverting the cross-correlation $\overline{\mathcal{C}}_\mu$ follow a very similar trend, separating slightly better the vase walls. This fact supports our claim of achieving sub-pixel accuracy directly at the original resolution. It also confirms two facts. First, the SS does not introduce unwanted artifacts to the reconstruction (the reconstruction is practically indistinguishable from the upsampled mean). Second, the method is very stable for a high number of iteration (although very slow in finding a final convergence). Inverting the average auto-correlation $\overline{\chi}_\mu$, instead, returns a reconstruction with a sharper PSF (panel D and corresponding plot) that comes along the inherent sub-pixel accuracy.

4 Analysis of the volume portion by averaging less views

In this chapter, we visualize the reconstruction results as a function of the number of views used: 2 opposite views taken at 180°, 4 orthogonal views in steps of 90°, 6 views in steps of 60°, and 12 views in steps of 30°. We do not show each single angle acquisition since it is in practice very similar to the result of 2 opposite views. We use the latter as a reference for a single acquisition.

Fig. 5 shows the results taking a slice along the tomographic direction in each of the cases considered. Along the tomographic plane, in general, the reconstruction visually increases in quality when fusing more views. On the other hand, along the planes parallel to the camera detection (Fig. 6, keeping the reference view at 0°) the quality seems to degrade with a higher number of views. However, this finds a balance along the direction perpendicular to the camera plane (Fig. 7).

To summarize, the effect of averaging multi-views is to balance the image quality along with the three-volume coordinates. Thus, the average PSF $\overline{h}$ becomes isotropic along every direction from which we observe the volume. This happens consistently for all the classes of reconstruction, and finding an optimal way of picking only the best frequencies along each direction is a common problem in multi-view fusion[3]. To find the best fusion mechanism (other than the mean) is not the focus of our study, here we want to assess that SS[2] and AU[4] reconstructions better resolve details if compared with standard averages. Being free from the alignment problem, we could carry out fusion protocols in the auto-correlation space to push the reconstruction method even further. We plan to investigate this in future developments of our protocol.

To assess the reconstruction performances for all the figures, we compare the first column (panels A, D, H, and M) with the second (B, E, I, and N) and third (C, G, L, and O). The results of the SS (and AU) always outperform those of the aligned means by exhibiting a sharper PSF and more contrasted images. The same considerations apply for the maximum intensity projections in Fig. 8, 9 and 10.

There is a final aspect to consider in this analysis. If we average 2 opposite views of the specimen, the SS does not exhibit any visible improvement over the simple mean. The explanation is simple and can be described in simple

formulas. Since, for opposite views the PSFs are identical, and thus $h^{0^\circ} = h^{180^\circ}$, we have that:

$$\begin{aligned}\overline{h} \star \overline{h} &= \left(h^0 + h^{180}\right) \star \left(h^0 + h^{180}\right) \\ &= \left(h^0 + h^0\right) \star \left(h^0 + h^0\right) \\ &= \left(2\, h^0\right) \star \left(2\, h^0\right) \\ &= 4\, h^0 \star h^0 = 4\, \mathcal{H}^0 \end{aligned} \tag{15}$$

On the other hand, for the auto-correlation average, we have that:

$$h^0 \star h^0 + h^{180} \star h^{180} = 2\, h^0 \star h^0 = 2\, \mathcal{H}^0 \equiv \overline{\mathcal{H}} \tag{16}$$

Thus, except for a trivial normalization term, $\overline{h} \star \overline{h}$ equals $\overline{\mathcal{H}}$ so that the result of SS is identical to that of the average mean (without the need for the alignment).

Fundings

H2020 Marie Skłodowska-Curie Actions (HI-PHRET project, 799230); H2020 Laserlab Europe V (871124).

Acknowledgment

Sample provided by Prof. J. Stein at the University of Bern and imaged by Jim Swoger at the Center for Genomic Regulation (CRG), Barcelona. The authors further thank Dr. Gianmaria Calisesi for inspiring discussions.

Competing interests

The authors declare no competing interests.

Materials & Correspondence

Correspondence to Daniele Ancora.

References

1. Guizar-Sicairos, M., Thurman, S. T. & Fienup, J. R. Efficient subpixel image registration algorithms. *Opt. letters* **33**, 156–158 (2008).

2. Schulz, T. J. & Snyder, D. L. Imaging a randomly moving object from quantum-limited data: applications to image recovery from second-and third-order autocorrelations. *JOSA A* **8**, 801–807 (1991).

3. Preibisch, S. *et al.* Efficient bayesian-based multiview deconvolution. *Nat. methods* **11**, 645 (2014).

4. Ancora, D. & Bassi, A. Deconvolved image restoration from auto-correlations. *IEEE Transactions on Image Process.* **30**, 1332–1341 (2020).

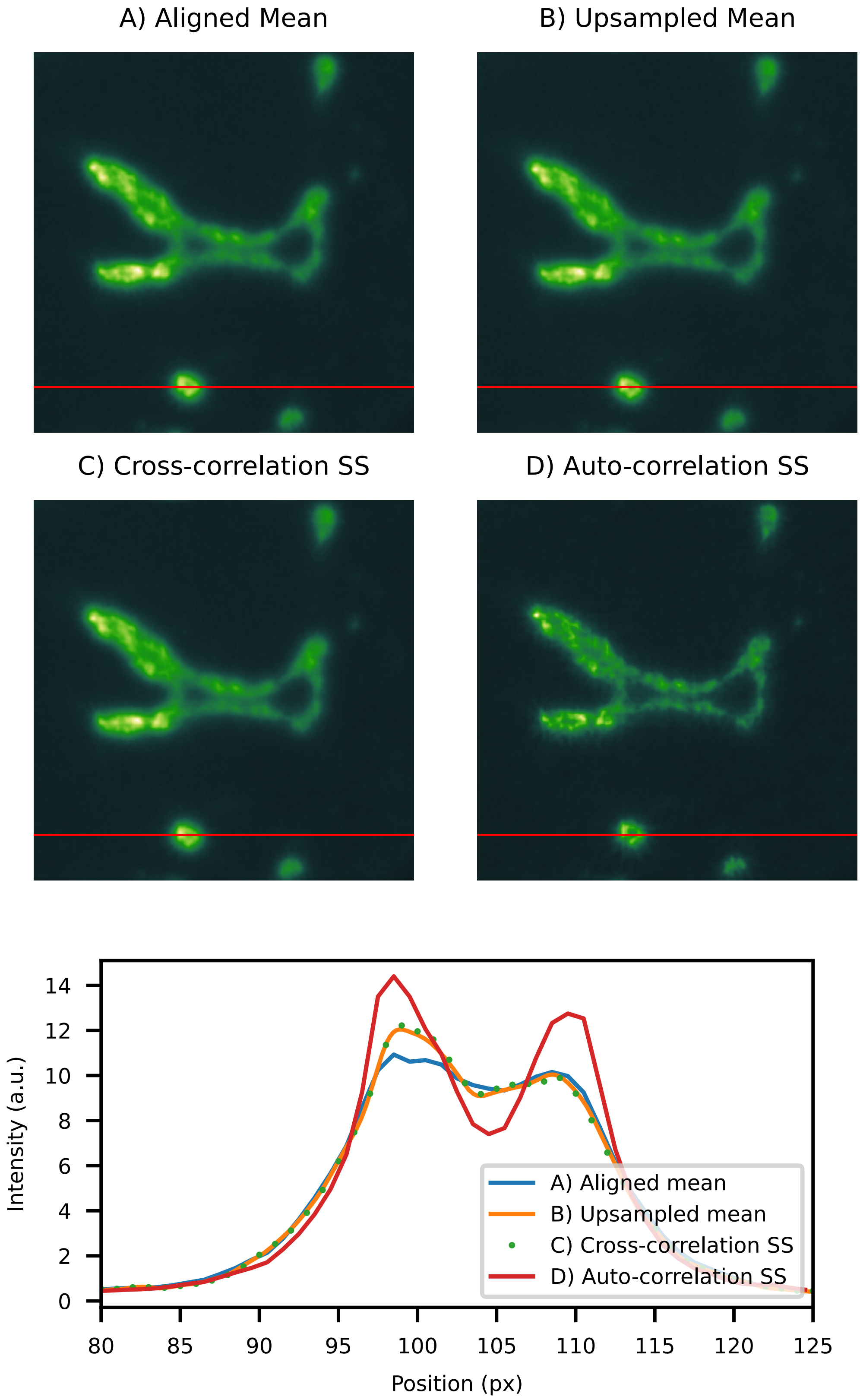

Figure 4. Assessing sub-pixel accuracy of the auto-correlation inversion with SS. A) The standard reconstruction image obtained by the aligned mean of 12 views. B) Upsampled by 10 times and aligned views, reconstruction accurate at 1/10 of a pixel. C) Reconstruction obtained averaging all the cross-correlation terms and inverting via SS. D) Reconstruction obtained averaging only the auto-correlations and inverting via SS.

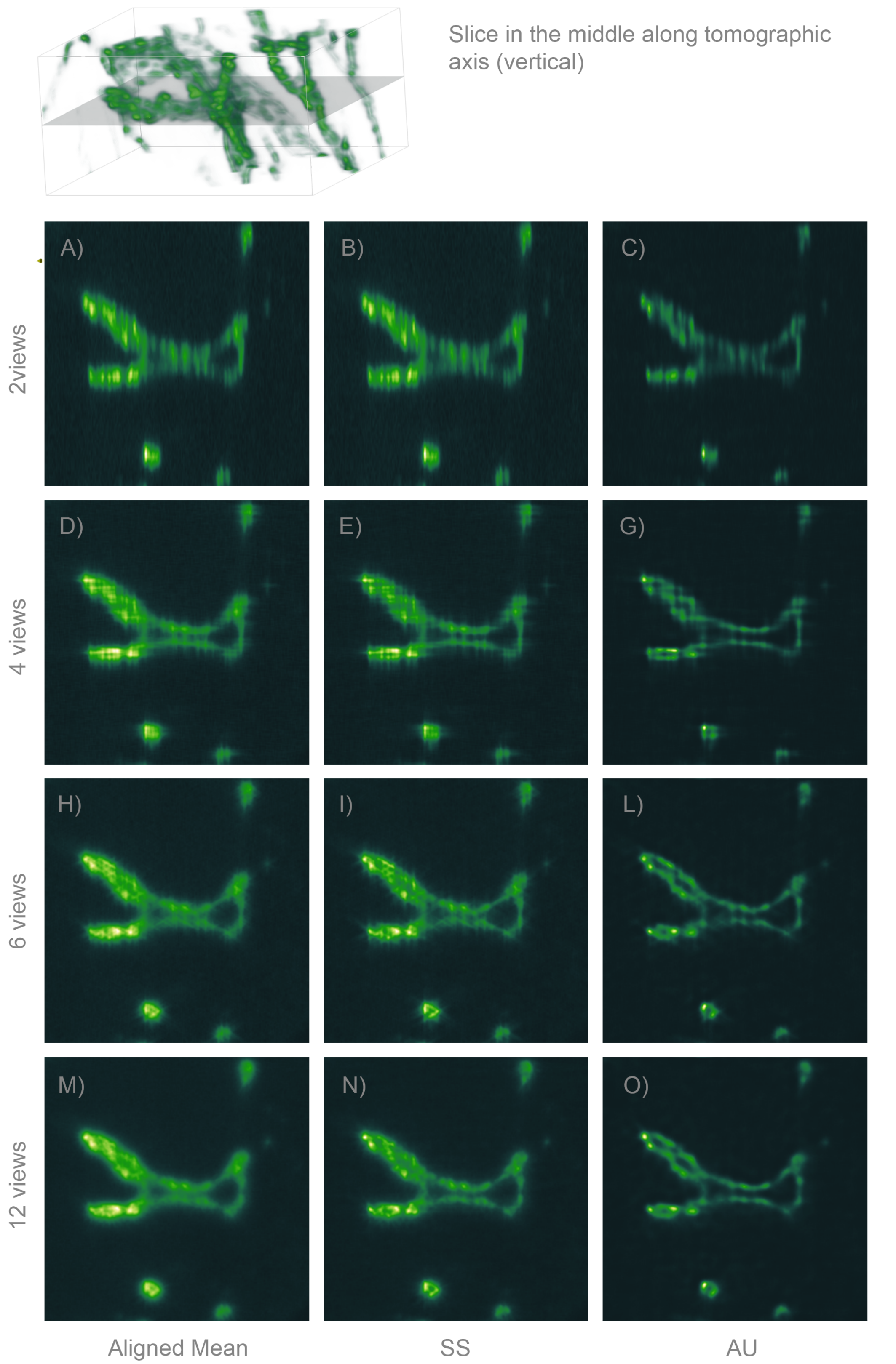

Figure 5. Tomographic slice through the cropped volume. A) Mean of two opposite views separated by 180°. B) SS reconstruction of 2 views. C) AU reconstruction of 2 views. D) Mean of four orthogonal views separated by 90°. E) SS reconstruction of 4 views. G) AU reconstruction of 4 views. H) Mean of 6 views with angular separation of $\varphi = 60°$. I) SS reconstruction of 6 views. L) AU reconstruction of 6 views. M) Mean of 12 views with angular separation of $\varphi = 30°$. N) SS reconstruction of 12 views. O) AU reconstruction of 12 views.

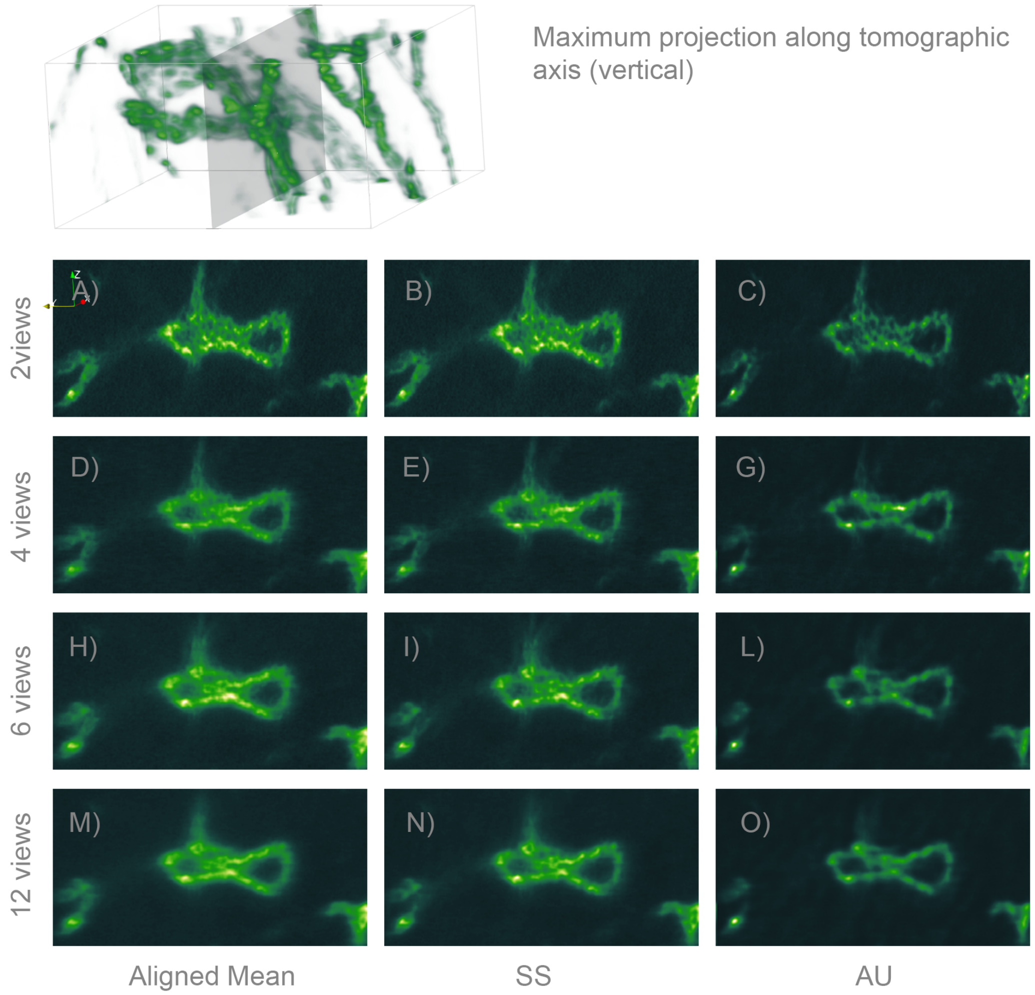

Figure 6. Slice through the cropped volume parallel to the camera detection plane of the reference $\varphi = 0°$. A) Mean of 2 opposite views separated by 180°. B) SS reconstruction of 2 views. C) Deconvolution with AU of 2 views. D) Mean of four orthogonal views separated by 90°. E) SS reconstruction of 4 views. G) AU reconstruction of 4 views. H) Mean of 6 views with angular separation of $\varphi = 60°$. I) SS reconstruction of 6 views. L) AU reconstruction of 6 views. M) Mean of 12 views with angular separation of $\varphi = 30°$. N) SS reconstruction of 12 views. O) AU reconstruction of 12 views.

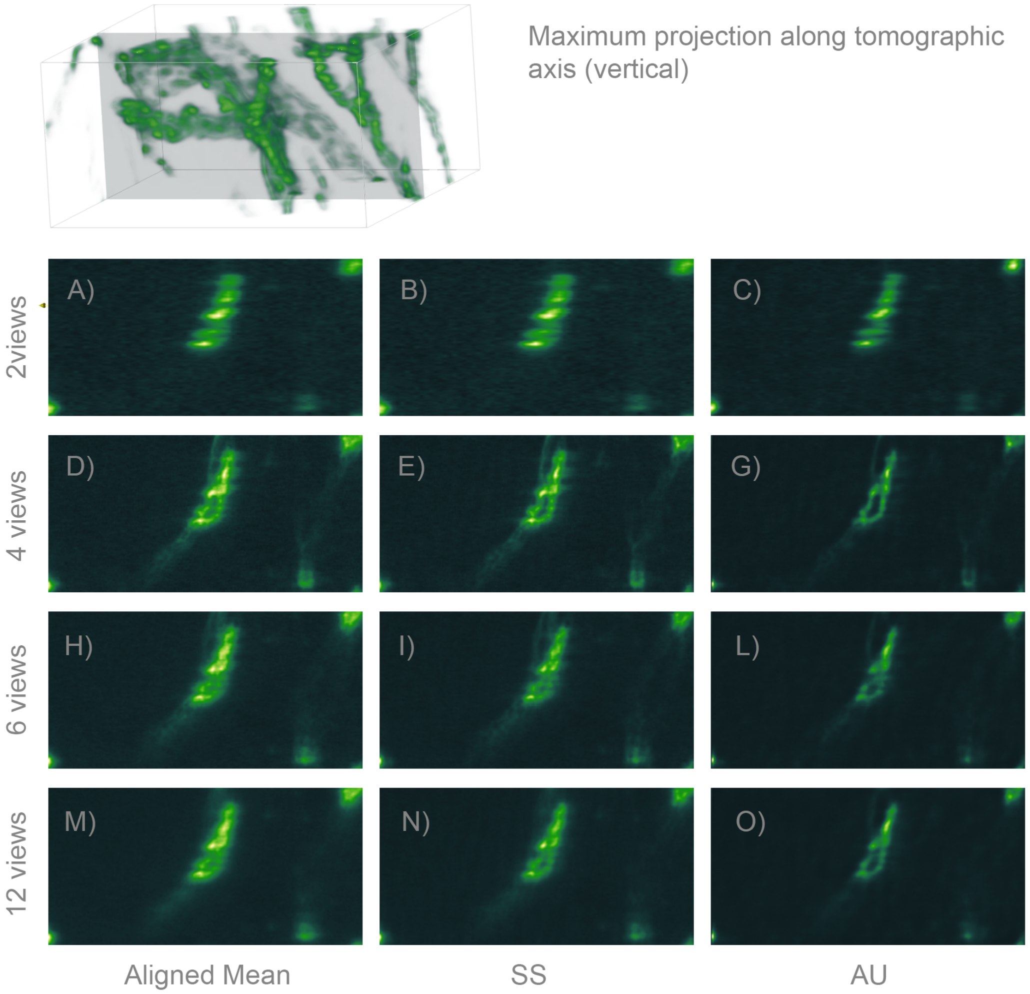

Figure 7. Slice through the cropped volume perpendicular to the camera detection plane of the reference $\varphi = 0°$. We notice the degradation in the image quality along the scanning axis due to lower accuracy. A) Mean of two opposite views separated by 180°. B) SS reconstruction of 2 views. C) AU reconstruction of 2 views. D) Mean of four orthogonal views separated by 90°. E) SS reconstruction of 4 views. G) AU reconstruction of 4 views. H) Mean of 6 views with angular separation of $\varphi = 60°$. I) SS reconstruction of 6 views. L) AU reconstruction of 6 views. M) Mean of 12 views with angular separation of $\varphi = 30°$. N) SS reconstruction of 12 views. O) AU reconstruction of 12 views.

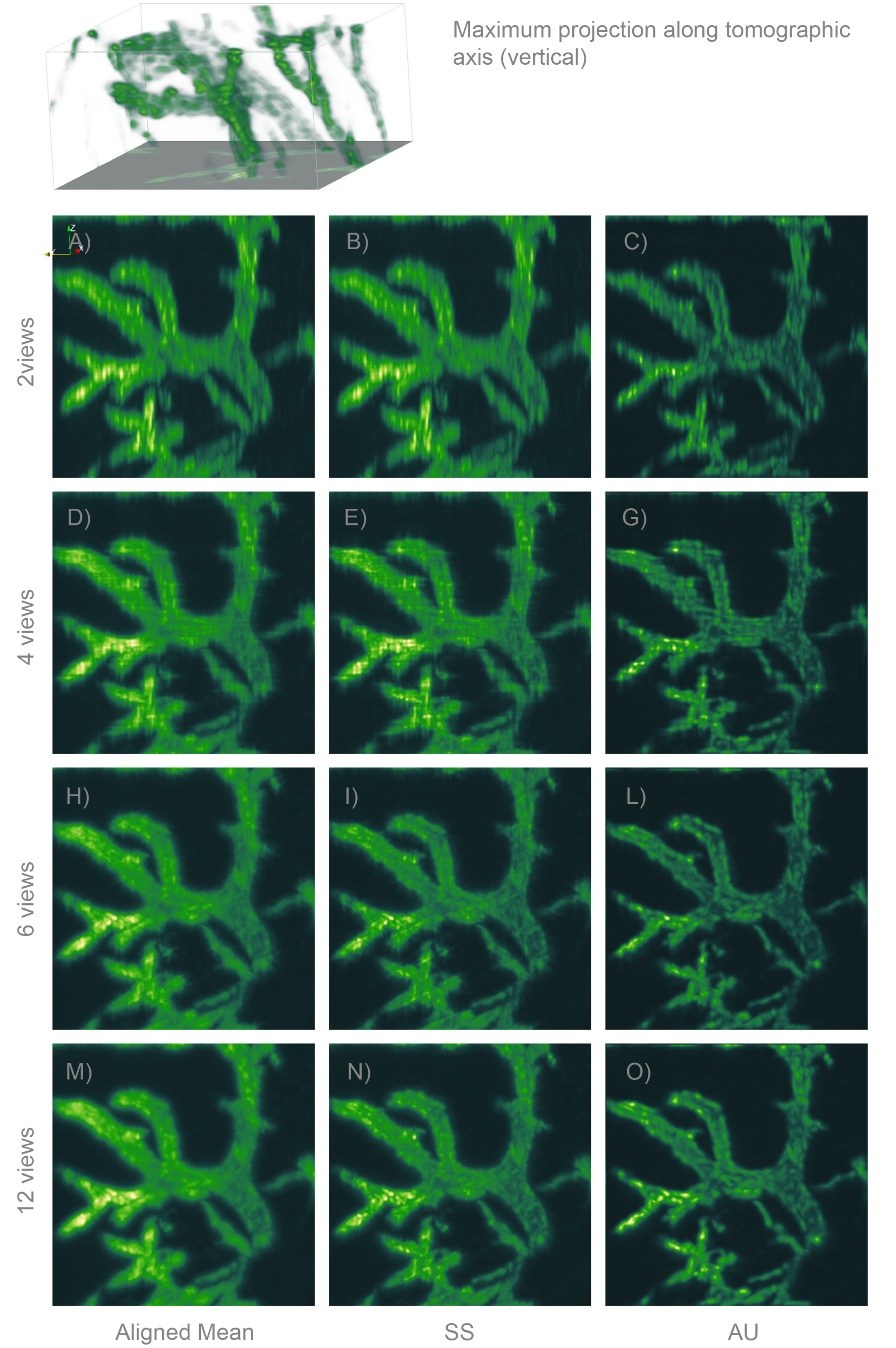

Figure 8. Maximum intensity projection (MIP) of the cropped volume along the tomographic axis. A) Mean of two opposite views separated by 180°. B) SS reconstruction of 2 views. C) AU reconstruction of 2 views. D) Mean of four orthogonal views separated by 90°. E) SS reconstruction of 4 views. G) AU reconstruction of 4 views. H) Mean of 6 views with angular separation of $\varphi = 60°$. I) SS reconstruction of 6 views. L) AU reconstruction of 6 views. M) Mean of 12 views with angular separation of $\varphi = 30°$. N) SS reconstruction of 12 views. O) AU reconstruction of 12 views.

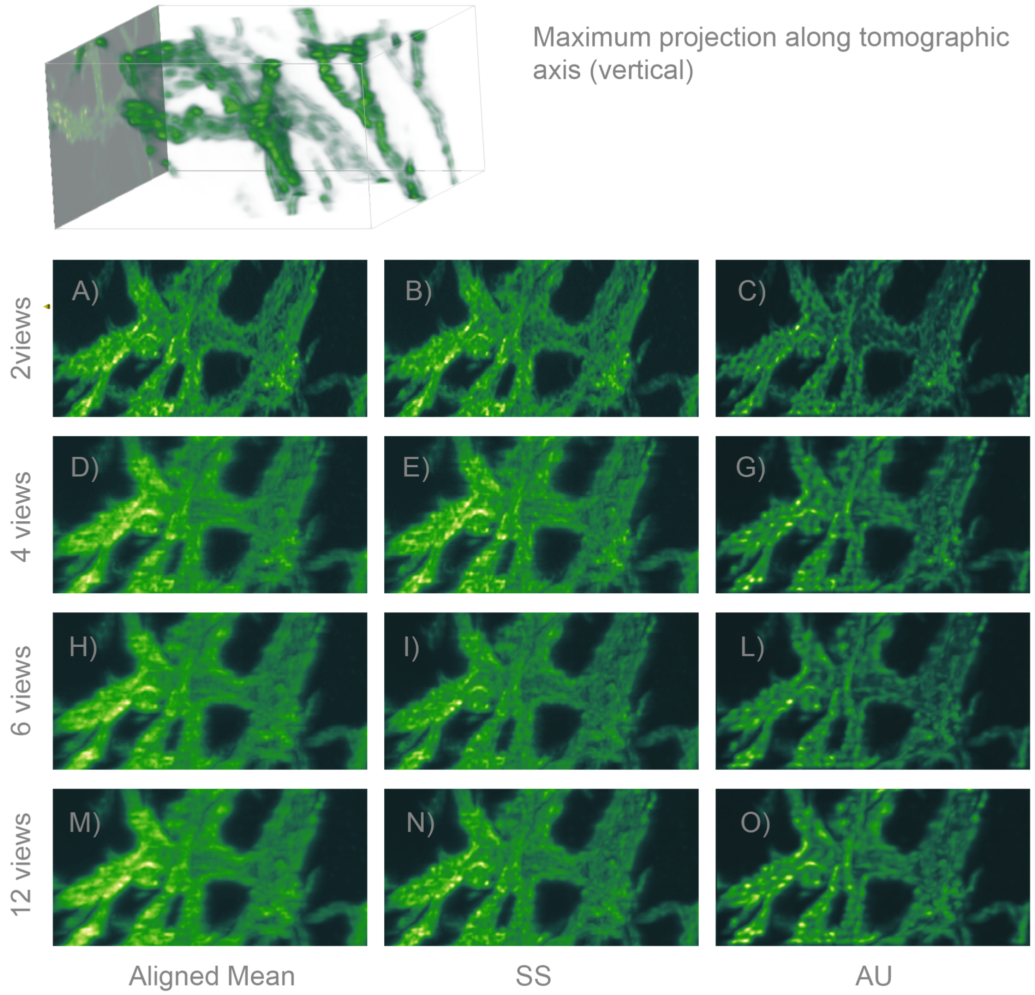

Figure 9. MIP of the cropped volume parallel to the camera detection plane of the reference $\varphi = 0°$. A) Mean of two opposite views separated by 180°. B) SS reconstruction of 2 views. C) AU reconstruction of 2 views. D) Mean of four orthogonal views separated by 90°. E) SS reconstruction of 4 views. G) AU reconstruction of 4 views. H) Mean of 6 views with angular separation of $\varphi = 60°$. I) SS reconstruction of 6 views. L) AU reconstruction of 6 views. M) Mean of 12 views with angular separation of $\varphi = 30°$. N) SS reconstruction of 12 views. O) AU reconstruction of 12 views.

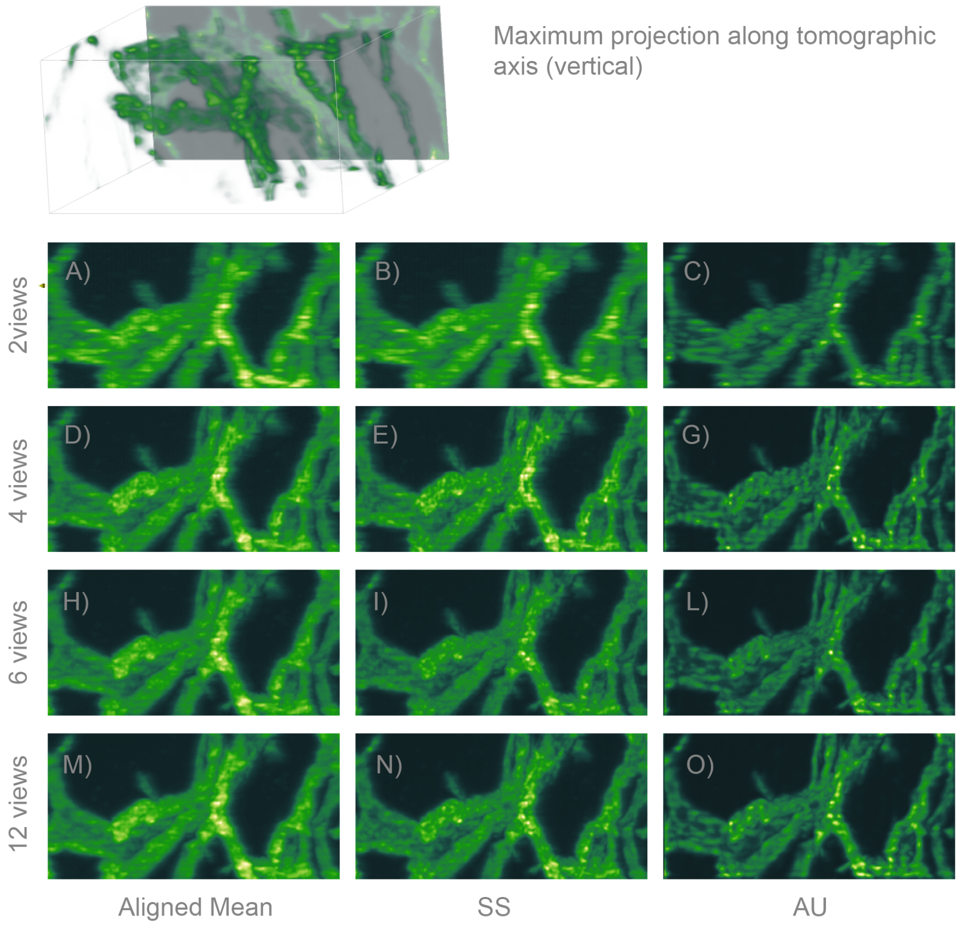

Figure 10. MIP of the cropped volume perpendicular to the camera detection plane of the reference $\varphi = 0°$. A) Mean of two opposite views separated by 180°. B) SS reconstruction of 2 views. C) AU reconstruction of 2 views. D) Mean of four orthogonal views separated by 90°. E) SS reconstruction of 4 views. G) AU reconstruction of 4 views. H) Mean of 6 views with angular separation of $\varphi = 60°$. I) SS reconstruction of 6 views. L) AU reconstruction of 6 views. M) Mean of 12 views with angular separation of $\varphi = 30°$. N) SS reconstruction of 12 views. O) AU reconstruction of 12 views.
